# The Effect of Very-Long-Chain *n*-3 Polyunsaturated Fatty Acids in the Central Nervous System and Their Potential Benefits for Treating Alcohol Use Disorder: Reviewing Pre-Clinical and Clinical Data

**DOI:** 10.3390/nu15132993

**Published:** 2023-06-30

**Authors:** Francisca Carvajal, Ainhoa Sánchez-Gil, Diana Cardona, Miguel Angel Rincón-Cervera, Jose Manuel Lerma-Cabrera

**Affiliations:** 1 Department of Psychology, University of Almeria, 04120 Almeria, Spain; maria.carvajal@ual.es (F.C.); asg837@ual.es (A.S.-G.); 2 Health Research Center, University of Almeria, 04120 Almeria, Spain; dcardona@ual.es; 3 Department of Nursing, Physiotherapy and Medicine, University of Almeria, 04120 Almeria, Spain; 4 Food Technology Division, ceiA3, CIAMBITAL, University of Almeria, 04120 Almeria, Spain; marincer@inta.uchile.cl; 5 Institute of Nutrition and Food Technology, University of Chile, Santiago 830490, Chile

**Keywords:** *n*-3 PUFA, DHA, EPA, DPA, alcohol use disorder, nutraceutical compound

## Abstract

Alcohol use poses a significant global health concern, leading to serious physical and socioeconomic issues worldwide. The current treatment options for problematic alcohol consumption are limited, leading to the exploration of alternative approaches, such as nutraceuticals. One promising target is very-long-chain *n*-3 polyunsaturated fatty acids (VLC *n*-3 PUFAs). This review aims to compile the most relevant pre-clinical and clinical evidence on the effect of VLC *n*-3 PUFAs on alcohol use disorders and related outcomes. The findings suggest that VLC *n*-3 PUFAs may alleviate the physiological changes induced by alcohol consumption, including neuroinflammation and neurotransmitter dysregulation. Additionally, they can reduce withdrawal symptoms, improve mood, and reduce stress level, all of which are closely associated with problematic alcohol consumption. However, more research is required to fully understand the precise mechanisms by which VLC *n*-3 PUFAs exert their function. Furthermore, PUFAs should not be considered a standalone solution, but as a complement to other therapeutic approaches. Although preliminary evidence supports the potential therapeutic effect of VLC *n*-3 PUFAs on problematic alcohol consumption, additional research is needed to validate these findings and determine the optimal use of PUFAs as part of a comprehensive approach to the treatment of alcohol use disorders.

## 1. Introduction

Among all the drugs of abuse, alcohol is the most widely used and abused worldwide. It is estimated that 43% of the population are current drinkers, with an individual average consumption of approximately 6.4 L of pure alcohol per year [1]. Alcohol consumption caused 1.78 million deaths in 2020 in addition to being a causal factor in more than 200 disease and injury conditions, including cardiovascular diseases, liver diseases, and type-2 diabetes [2]. In fact, alcohol misuse is a leading risk factor for death and disability in people aged 15–39 years [3]. 

In addition to the level of consumption, drinking patterns are also relevant to health. Thus, alcohol consumption, particularly heavier drinking (defined as 60 or more grams of pure alcohol on at least one single occasion at least once per month), has serious health consequences [4]. Alcohol-related costs to government finances are estimated to exceed 2.5% of the gross domestic product [5]. In addition, it has been shown that this pattern of consumption can increase an individual’s risk of alcohol use disorder (AUD) [6]. These harmful effects of heavy drinking are of particular concern in the adolescent population, with one out of eight adolescents showing this pattern of consumption [1]. Clinical and pre-clinical studies demonstrate that adolescents are particularly susceptible to the detrimental effects of alcohol. In particular, the young adolescent brain shows a greater sensitivity to alcohol-induced brain damage compared with the adult brain [7]. Heavy alcohol consumption during adolescence is associated with cognitive, neurobiological, and behavioral alterations that persist into adulthood and with an increased risk of developing alcohol dependence in the future [8].

For this reason, a global strategy to reduce the harmful use of alcohol is necessary, not only to decrease the negative consequences of drinking and alcohol intoxication but also to prevent the initiation of drinking among children and adolescents [1]. Great advances have been made in understanding the neuroscience of drug addiction, facilitating the exploration of new pharmacological targets in the treatment of this chronic disease. However, promising pre-clinical results have failed to translate into novel treatments. In the particular case of excessive alcohol consumption, the therapeutic options to help people with problematic alcohol consumption achieve abstinence or reduce their alcohol consumption are rather limited and have a small effect size [9]. Therefore, finding ways to improve the treatment of alcoholism poses a major challenge. 

The emerging evidence suggests that activation of the innate immune system may be a key factor in the effects of alcohol not only on the central nervous system (CNS) but also on the cognitive and behavioral alterations associated with heavy drinking [10]. Given that the use of nutraceutical intervention has been recognized as a promising option for the prevention of a wide range of neuroinflammation-related mental disorders [11], it has recently been proposed as a complementary model to pharmacological and behavioral treatments against drug addiction. The use of compounds from food sources has received considerable interest because they can provide health benefits or protection against chronic disease with minimal side effects (for a review, see [12]). Prebiotics, probiotics, dietary fibers, or dietary supplements are some of the compounds considered as nutraceuticals. In the present review, we focus on showing the therapeutic potential of polyunsaturated fatty acids from the *n*-3 family, specifically, very-long-chain *n*-3 polyunsaturated fatty acids (i.e., polyunsaturated fatty acids with more than 20 carbons; VLC *n*-3 PUFAs). VLC *n*-3 PUFAs, such as docosahexaenoic, eicosapentaenoic, and docosapentaenoic acids (DHA, EPA, and DPA, respectively) are nutritionally important for humans and many other animals. In recent years, VLC *n*-3 PUFAs have attracted increasing interest because of their pivotal roles in the inflammatory response. Furthermore, given that neuroinflammation has been shown to contribute to the development of drug addiction, the therapeutic potential of PUFAs in substance abuse has been proposed [13]. Therefore, the focus of this work is to summarize recently published articles analyzing the role of VLC-PUFAs in alcohol use disorders and their related outcomes. We show evidence suggesting that VLC *n*-3 PUFAs may alleviate physiological (neuroinflammation, neurotransmitter dysregulation, etc.) and behavioral changes (e.g., impulsivity or negative emotional state) induced by alcohol consumption. Taking all these data together, VLC *n*-3 PUFAs appear to present a promising therapeutic strategy for treating alcohol use disorders and their related outcomes.

## 2. Methodology

In March 2023, a comprehensive literature search was conducted in databases including Pubmed, Science Direct, and Google Scholar. The search strategy employed a combination of natural language (“PUFA”, “polyunsaturated fatty acids”, “neuroinflammation”, “cognition”, “emotion”, and “alcohol use disorder”) and structured language using the MeSH descriptors (“fatty acids, Omega-3”, “neuroinflammatory diseases”, “cognition”, and “alcohol-related disorders”). Each article was selected for relevance based on title and abstract, and studies that did not investigate the effect of VLC *n*-3 PUFAs on neuroinflammation, behavior, cognition, and/or alcohol-related disorder were excluded. The authors, who independently conducted the study selection, extracted pertinent data from selected studies, and the results were thoroughly discussed to develop the current comprehensive review. 

## 3. General Overview of Very-Long-Chain *n*-3 Polyunsaturated Fatty Acids

### 3.1. Structure, Health Effect, and Intake Recommendation for VLC n-3 PUFAs

**Figure 1:**
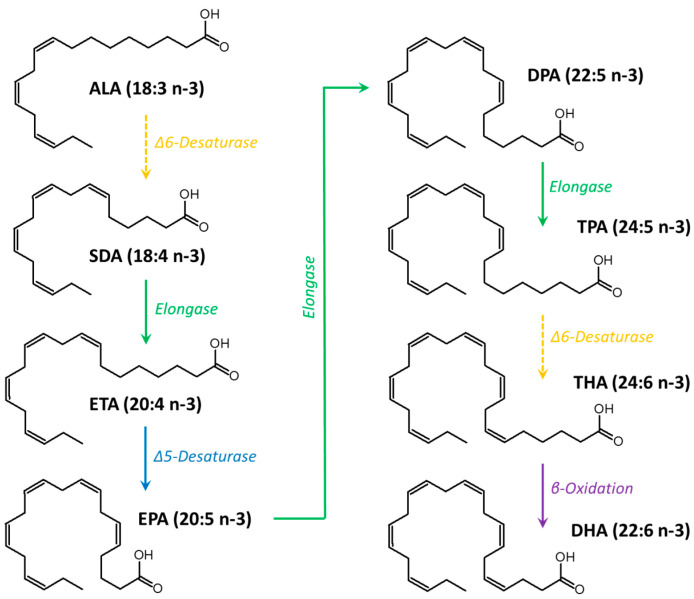
Metabolic pathway of *n*-3 PUFA from α-linolenic acid (ALA), which is provided by the diet, to docosahexaenoic acid (DHA). SDA: stearidonic acid; ETA: eicosatetraenoic acid; EPA: eicosapentaenoic acid; DPA: docosapentaenoic acid; TPA: tetracosapentaenoic acid; THA: tetracosahexaenoic acid.

Fatty acids (FAs) are organic molecules that can be classified based on the number of double bonds (unsaturation) in their hydrocarbon chain. FAs containing no unsaturation are called saturated FA (SFA), those containing a single unsaturation are called monounsaturated FA (MUFA), and those with two or more double bonds in their hydrocarbon chain are called polyunsaturated fatty acids (PUFAs). Among PUFAs, the *n*-3 or omega-3 family is widely recognized for its fundamental role in the human organism. Within *n*-3 PUFAs, α-linolenic acid (ALA, 18:3 *n*-3) is an essential PUFA that cannot be synthesized endogenously and must be provided by the diet. Once ingested, ALA is converted by the metabolic pathway to an *n*-3 PUFA with a longer chain, also known as a very-long-chain *n*-3 PUFA (VLC *n*-3 PUFA) [14]. Eicosapentaenoic acid (EPA, 20:5 *n*-3), docosapentaenoic acid (DPA, 22:5 *n*-3), and docosahexaenoic acid (DHA, 22:6 *n*-3) are VLC *n*-3 PUFAs that can be considered “conditionally essential” due to their low metabolic conversion efficiency of ALA because of the limited activity of the Δ6-desaturase in humans (Figure 1) [14,15]. Therefore, it is generally recommended to consume VLC *n*-3 PUFAs in the diet to reach their required physiological levels.

EPA and DHA are the two most studied VLC *n*-3 PUFAs with regard to their beneficial effects on the human organism, although emerging evidence suggests a relevant role for DPA as well. FAs are located in the phospholipids of cell membranes and contribute to membrane fluidity, cell signaling, and the function of membrane proteins [16]. The consumption of EPA and DHA has been associated with a lower risk of cardiovascular diseases (CVDs), rheumatoid arthritis, blood pressure issues, and cancer; the prevention of psychological and behavioral disorders and cognitive decline; the regulation of gene expression; and the reduction of harmful effects associated with fatty liver and oxidative stress [14,15,16,17,18,19]. They are metabolic precursors of anti-inflammatory lipid mediators (eicosanoids and docosanoids, respectively) [16,20]. DHA plays a particularly important role in the development and function of the neurological and visual systems [21,22]. Neuroprotectin D1 and other specialized DHA-derived pro-resolving lipid mediators have been shown to exert neuroprotective activity by preserving the physiology of neurons and glial cells [22,23].

DPA has been studied much less than EPA and DHA in terms of its health effects, likely due to the difficulty of obtaining purified DPA (>95%) at an affordable price for clinical and pre-clinical assays, either through purification from natural sources (as DPA is less abundant than EPA and DHA in nature) or chemical synthesis. However, current existing evidence suggests that *n*-3 DPA may contribute to the improvement of biomarkers related to cardiovascular and metabolic disorders; additionally, its roles as a neuroprotector in the elderly and in modulating inflammation through DPA-derived docosanoids have been pointed out [24,25]. However, more research is needed to consistently confirm the effects of DPA in the human organism. 

The Food and Agriculture Organization (FAO) recommends a daily intake of 250 mg of EPA and DHA for adult males and non-pregnant/non-lactating women, and 300 mg of EPA and DHA for pregnant/lactating women, of which at least 200 mg should be DHA. The FAO has also established the upper level of acceptable daily consumption at 2 g of EPA and DHA considering the sustainability criteria regarding the excessive strain on fish stocks (the main source of VLC *n*-3 PUFA), although no adverse effects were reported at higher intakes (up to 3 g/day) in short- and intermediate-term randomized trials [26]. DPA was not included in the recommendation due to the limited existing evidence on this VLC *n*-3 PUFA. 

The European Food Safety Authority (EFSA) also established a value of 250 mg of EPA and DHA as the recommended daily intake of both *n*-3 PUFA to reduce the risk of heart disease in healthy adults (primary prevention). Furthermore, pregnant or lactating women should consume an additional 100 to 200 mg of preformed DHA daily to compensate for the transfer of DHA to the fetus/infant [27]. The EFSA Panel stated that supplemental intakes of EPA alone, DHA alone, and EPA and DHA at doses up to 1.8, 1, and 5 g/day, respectively, do not raise safety concerns for the adult general population. Data are not available for DPA when consumed alone.

The International Society for the Study of Fatty Acids and Lipids (ISSFAL) recommends a minimum daily intake of 500 mg of EPA and DHA for good cardiovascular health in the general adult population, while pregnant and lactating women should consume at least 200 mg/day of DHA. The ISSFAL also established that daily supplementation with 1000 mg of EPA and DHA is effective in reducing the risk of early birth for women with low base levels of *n*-3 PUFA, preferably if supplementation begins before 20 weeks of gestation [28].

The American Heart Association (AHA) recommends a daily intake of 1 g of EPA and DHA for the secondary prevention of coronary heart disease (CHD) and a daily intake of 2 to 3 g/day to help lower blood pressure [11].

The Global Organization for EPA and DHA Omega-3 (GOED) has established the following recommendations for daily intakes of EPA and DHA: (i) 500 mg for the general healthy adult population to reduce the risk of CHD; (ii) 700 mg for pregnant and lactating women, with at least 300 mg of DHA; (iii) 1 g/day for secondary prevention of CHD; (iv) intakes greater than 1 g for a variety of health conditions, such as high blood pressure or hypertriglyceridemia [29].

### 3.2. Sources of VLC n-3 PUFAs

Foods from marine origin are the main dietary sources of VLC *n*-3 PUFAs (Table 1). Typically, consuming two or three servings of marine foods per week is enough to reach the recommended intake of EPA and DHA (250–500 mg/day). However, as a result of the increasing strain on marine resources and concerns about sustainability, alternative sources have emerged for the production of EPA and DHA oils and concentrates. These alternative sources are commonly used in the formulation of dietary supplements or nutraceuticals. EPA and/or DHA production has been reported from microorganisms, such as microalgae (e.g., *Amphidinium* sp., *Heterocapsa tricuetra*, *Isochrysis galbana*, *Pavlova gyrans*, *Prorocentrum micans*, *and Nannochloropsis* sp.), fungi (e.g., *Aurantiochytrium* sp., *Mortierella alpina*, *Schizochytrium* sp., *Thraustochytrium* sp., and *Crypthecodinium cohnii*), and fish by-products [30,31,32].

## 4. Effect of VLC *n*-3 PUFAs on the Central Nervous System (CNS)

### 4.1. Physiological Functions of VLC n-3 PUFAs on the CNS

The role of VLC *n*-3 PUFAs in the development and function of the CNS and its neuroprotective effects have been evidenced in previous works. Among the VLC *n*-3 PUFAs, DHA has been particularly studied in terms of its relationship with brain functions. In fact, DHA is one of the most abundant FAs in the brain structural lipids, and it plays an essential role in neuronal growth, cell signaling, synthesis of pro-resolving lipid mediators, and synaptic plasticity [16]. It has been shown that a low intake of DHA during pregnancy may lead to abnormal development and function of the CNS and that a DHA supply may improve behavioral symptoms related to neuropsychiatric disorders at the mild stage [42,43]. Neuronal cells cannot endogenously synthesize VLC *n*-3 PUFAs and must be provided from the diet. Thus, nutrition is a relevant factor in the FA profile in the brain. Once uptaken, VLC *n*-3 PUFAs are metabolized to specialized pro-resolving lipid mediators via cicloxygenases, lipoxygenases, and CYP450 monoxygenases [44].

DHA and DPA are located mainly in phosphatidylethanolamine (PE) and phosphatidylserine (PS), and EPA is found mainly in phosphatidylinositol (PI) [45]. DHA available on the brain cell membrane is involved in the modulation of PS synthesis, and an increase in PS in neuronal membranes may have an impact on the neuronal survival [45]. 

EPA and DHA are involved in neurogenesis, which is a mechanism of compensation for neuronal loss in the brains of mammals, giving place to neurons from neural stem cells. This mechanism is age-dependent, showing a slower rate and lower efficiency during adulthood than in younger individuals [46]. A low rate of hippocampal neurogenesis has been associated with depression [47] and the production of pro-inflammatory cytokines, such as interleukin 1β (IL-1β), which is related to impaired learning and memory capacities [47]. EPA and DHA showed protective effects against the detrimental influence of IL-1β on human hippocampal neurogenesis, likely due to their involvement in regulating IL-1β signaling by modulation of the kynurenine pathway [47]. However, most evidence regarding understanding the regulation of hippocampal neurogenesis has been obtained using animal models, and data from human studies are still scarce [48].

### 4.2. Effect of VLC n-3 PUFAs on Neuroinflammation

Among the functions that VLC *n*-3 PUFA and its mediators have in regulating various processes within the brain, their effect on the neuroinflammatory response has attracted a lot of interest in recent years [44,49]. Perhaps this interest is partly motivated by the fact that neuroinflammation is involved in contributing to a variety of neurological diseases, including neurodegenerative disorders, neuroimmune diseases, and cerebrovascular disorders [50]. Neuroinflammation involves the activation of microglia and astrocytes, which promotes the release of various cytokines, such as IL-1β, IL-6, and TNF-α, and the generation of reactive oxygen species that alter homeostasis and can cause adverse effects on the CNS [50]. Because alleviation of neuroinflammation is believed to reduce disease severity and delay the progression of disease, studying the precise neuroinflammatory mechanism in which VLC *n*-3 PUFAs are involved could facilitate a novel therapeutic approach for these diseases. 

**Figure 2:**
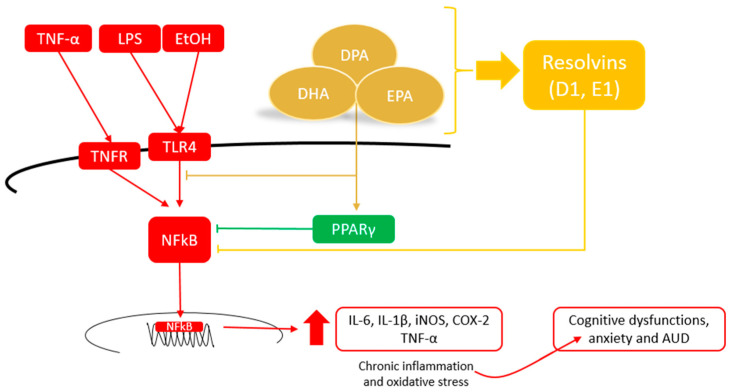
Potential mechanisms of VLC *n*-3 PUFAs to alleviate neuroinflammation and contribute to neuroprotective functions.

Although the exact mechanism underlying their mode of action has not yet been established, VLC *n*-3 PUFAs can regulate this response by inhibiting the activation of pro-inflammatory pathways (e.g., activation of peroxisome proliferator-activated receptors, and PPARs) and reducing the expression of cytokines (mainly IL-6 and TNF-α) [44,49]. Furthermore, it has been proposed that an anti-inflammatory status may be mediated by the production of specialized mediators, such as resolvins (RVs) and protectins (PD) [51,52,53] (Figure 2). Taken together, these studies suggest that the supply of VLC *n*-3 PUFAs could become a suitable strategy to resolve brain inflammation and contribute to neuroprotective functions for brain inflammatory diseases. In the following paragraphs, the latest results on this subject from in vitro and in vivo studies are shown in detail (Table 2).

To start with, several in vitro studies have revealed that VLC *n*-3 PUFA and its derivatives have an anti-inflammatory effect on the brain. Studies have shown that DHA-phospholipids downregulate the expression of mRNA for IL-6 and IL-1 β mRNA induced by lipopolysaccharide (LPS) in the immortalised microglial cell line BV2 [54]. Furthermore, free DHA has been found to reduce LPS-induced levels of IL-1β, IL-6, and TNFα through its effect on LPS receptors and the signalling pathway [55]. Similar results have been observed in primary bovine mammary epithelial cells [56]. In addition, DHA-enriched astrocytes showed a considerably reduced response to IL-1β, a decrease in iNOS and COX-2 levels, and a reduction in pro-inflammatory TNF-α and IL-6 release [57]. EPA has also been identified as having an anti-inflammatory effect [58,59]. EPA inhibits the production of the pro-inflammatory cytokine IL-6 and downregulates its expression at the mRNA level in IL-1β-stimulated C6 glioma cells in a dose-dependent manner [59]. This effect is blocked by a peroxisome proliferator-activated receptor (PPAR) antagonist, suggesting that PPAR signalling is necessary for the anti-inflammatory effect of EPA [59]. Both DHA and EPA are potent activators of PPAR, and recent research has suggested that they can inhibit neuroinflammation by blocking the activation of NF-κB, a pro-inflammatory transcription factor [60]. In terms of the mechanisms of action of PUFAs, some studies have shown that EPA and DHA suppress the production of IL-6 and TNF-α by LPS-stimulated MG6 microglial cells by activating SIRT1 pathways [61]. Other research has indicated that DHA, but not EPA administration, leads to a downregulation of CD14 and toll-like receptor 4 (TLR4) cell-surface expression in LPS-stimulated BV-2 microglial cells, suggesting that DHA targets the surface location of LPS receptor [55]. Finally, studies carried out with docosapentaenoic acid (DPA) reported that treatment with this VLC *n*-3 PUFA reduced COX2 mRNA expression in amyloid-beta42 oligomer-challenged BV2 microglial cells [62] and decreased the TNF-α, IL-1β, and NO levels in LPS-stimulated BV2 cells [63]. It also downregulated the mRNA expression of pro-inflammatory factors, such as IL-1β, IL-6, iNOS, and COX-2, in LPS-activated murine macrophages, such as RAW264.7 cells, similar to the DHA treatment [64]. 

In the brain, VLC *n*-3 PUFAs have been found to exhibit anti-inflammatory activity through their indirect effect on microglia via the synthesis of a pro-resolving mediator of inflammation. In vitro studies have shown that RvD1 synthesized from DHA and RvE1 derived from EPA can inhibit the expression of pro-inflammatory cytokines in microglial cell cultures by pre-incubation of LPS-induced BV2 cells [52,53]. These studies have reported a significant reduction in the gene expression of TNF-α, IL-6, and IL-1β in response to resolvins [51]. Further studies have shown that the protective role of RvE1 against LPS-induced neuroinflammation appeared to be mediated by the regulation of NF-κB signalling pathways, while RvD1 regulated microRNA expression [51]. However, additional research is needed to clarify the mechanism of action of these compounds. In this respect, new approaches using supramolecular nanotheranostics could provide us with more information about the inflammatory effects of VLC *n*-3 PUFAs on the brain [65].

Animal studies have also shown the anti-inflammatory role of VLC *n*-3 PUFAs in the brain. For example, rodent studies have shown that the administration of VLC *n*-3 PUFAs protects against neuroinflammation caused by pro-inflammatory treatments, aging, or acute injury. These studies are consistent with the in vitro data indicating that increased PUFA level attenuates neuroinflammation after systemic LPS administration. In one study, peripheral administration of LPS to fat-1 transgenic mice with endogenously increased VLC *n*-3 PUFAs generated a lower production of pro-inflammatory mediators than in the wild-type littermates [66]. Pharmacological studies have also shown that central administration of DHA-phospholipid decreased LPS-induced IL-6 mRNA expression in the hippocampus of mice [54]. Furthermore, dietary EPA has been found to attenuate IL-1β-induced glial activation and the upregulation of TNF-α and to increase the expression of the brain-derived neurotrophic factor (BDNF) in the hippocampus of rats [58]. Similarly, EPA-enriched phospholipids have been found to reduce LPS-induced increases in the level of IL-1β in the hippocampus, thalamus, and cortex [67]. A recent study has shown that pups exposed to a maternal diet enriched with stearidonic acid (SDA) that increased total VLC *n*-3 PUFAs (EPA, DPA, and DHA) exhibited less inflammatory IL-6 and TNF-α in response to LPS than the control group [68]. 

In aged mice, a diet of DHA/EPA decreased the mRNA expression of TNF-α and IL-6 and protected against changes in the astrocyte morphology in the hippocampus [69]. Similarly, DPA reduced microglial activation, caspase-3, and oxidative stress in aged rats [70]. In mouse models of Alzheimer’s disease, DPA treatment reduced pro-inflammatory cytokines of IL-6, TNF- α, and COX2 mRNA expression, while increasing IL-10 [62].

In humans, lower levels of *n*-3 PUFAs have been associated with higher serum levels of IL-6 and TNF-α, according to epidemiological and observational studies [71]. Plasma inflammatory biomarkers have also been inversely associated with DPA levels [72]. Conversely, *n*-3 PUFA supplementation was able to modulate cytokines involved in inflammation [73,74,75,76]. For instance, DHA and EPA supplementation significantly reduced the IL-6 and TNF-α levels in older adults with mild cognitive impairment [73]. In patients with type 2 diabetes, supplementation with VLC *n*-3 PUFAs was found to decrease the TNF-α and c-reactive protein levels [76]. Additionally, *n*-3 PUFA supplementation decreased LPS-stimulated IL-6 and TNF-α production in healthy young adults [74] and decreased circulating inflammatory markers in adults [75]. 

### 4.3. Effect of VLC n-3 PUFAs on Cognitive and Behavioral Level

The last decade has witnessed the emergence of many studies aimed at evaluating the potential beneficial effect of PUFAs on cognitive and behavioral processes (see Table 2). Several works support the hypothesis that VLC *n*-3 PUFA plays a role in cognitive and behavioral processes. Thus, a dietary reduction in VLC *n*-3 PUFA intake in rodents has been associated with impairments in spatial learning, working memory, and olfactory discrimination learning [77,78]. On the contrary, VLC *n*-3 PUFA dietary supplementation can improve cognitive processes. Thus, it has been demonstrated that a PUFA-enriched diet improves memory deficit in rats [69,79], DHA markedly improves spatial memory in female Sprague–Dawley rats [80], and DPA attenuates age-related deficits in spatial learning and long-term potentiation in aged rats [70]. 

Taking into account these results, several clinical trials have proposed that VLC *n*-3 PUFA could play a protective role against age-related cognitive decline (for a review, see [81]). Several studies in middle-aged and elderly populations have shown that a higher proportion of *n*-3 PUFA in plasma is associated with a lower risk of cognitive decline [82,83,84]. Moreover, the beneficial effect of DHA supplementation on working and short-term memory has been reported in older adults with mild cognitive impairment (MCI) [73,85] and in healthy subjects [86]. Similarly, supplementation with DHA and EPA can also improve memory and mood in patients with mild–moderate Alzheimer’s disease [87].

Despite the promising beneficial effect of VLC *n*-3 PUFAs against cognitive decline, other studies have not shown a significant effect of supplementation with VLC *n*-3 PUFAs on cognitive function in elderly populations [88,89]. A recent meta-analysis of observational and experimental studies showed a beneficial effect of VLC *n*-3 PUFAs on the executive function of older people, rather than on their overall cognitive performance [90]. The considerable heterogeneity of the participants, differences in the cognitive measurement methods, and the widely disparate doses used are some aspects that need to be taken into account to explain these divergent results. To improve the generalization of results, these variables should be methodologically controlled in future research. 

It has also been established that a deficiency in *n*-3 PUFA over generations leads to substantial changes in the attentional processes and impulsive behavior in Wistar rats, as evaluated by the 3CSRTT [91]. The detrimental effect of transgenerational depletion of *n*-3 PUFA was partially corrected by the introduction of a sufficient diet of *n*-3 PUFA [91]. In a previous study, Dervola et al. (2012) found that *n*-3 PUFA supplementation significantly improved reinforcement-controlled attention and reduced lever-directed hyperactivity in spontaneously hypertensive rats (SHR), a genetically determined attention-deficit hyperactivity disorder (ADHD) model that is well validated [92]. The current evidence for the use of *n*-3 PUFAs as a treatment for ADHD in children is mixed. Although some studies indicated that *n*-3 PUFA supplementation is effective in children with attention deficit disorders [93,94,95], a recent meta-analysis has shown weak supporting evidence for the use of *n*-3 PUFAs to treat ADHD in children [11]. Given that self-reported impulsivity in non-patient samples is related to circulating levels of *n*-3 PUFAs [96,97], it is possible that the pre-existing deficiency influences the treatment efficacy. However, this hypothesis needs to be explored in future studies. 

The literature contains numerous studies investigating the potential of VLC *n*-3 PUFAs for preventing and treating mood disorders [98]. A growing body of evidence shows that individual differences in depression and anxiety symptoms are related to circulating levels of PUFAs. For example, a recent study demonstrated that exposition to chronic unpredictable mild stress (CUMS), which is a condition used to induce depression-like behaviors in animal models, is associated with lower brain levels of VLC *n*-3 PUFAs (EPA, DPA, and DHA) [99]. Several cross-sectional studies have found low plasma levels of *n*-3 PUFAs in patients with depressive disorders [100,101]. Additionally, baseline PUFA levels predict a subsequent response to standard antidepressants [102]. Furthermore, an adequate amount of VLC *n*-3 PUFA is associated with a lower risk of depression. Data from animal models have shown that dietary supplementation with *n*-3 PUFAs improves depressive symptoms in rodents [67] and seems to normalize mood state, acting as a mood-stabilizing agent in a KO mouse model of bipolar disorder [103]. Clinical studies have found that a higher intake of *n*-3 PUFAs among women in the United States is associated with lower amounts of depressive symptoms [104]. A longitudinal study carried out in Japan found that a higher intake of VLC *n*-3 PUFAs is associated with a lower risk of depressive symptoms [105]. Therefore, the adjunctive use of *n*-3 PUFAs is recommended for the treatment of major depressive disorders [11], particularly in people with elevated inflammatory markers or in cases of dietary deficiency [106]. 

Studies on rodents have shown that long-term deficiency of VLC *n*-3 PUFAs causes changes in the dopaminergic and serotoninergic systems in the brain [107], suggesting that this may be one of the neurobiological mechanisms underlying the antidepressant effect of PUFAs. Several works have also proposed that, since depression has been associated with activation of the inflammatory response, another possible mechanism by which PUFA alleviates depression could be mediated by its anti-inflammatory response [67,108]. Interestingly, EPA and DHA supplementation reduced inflammatory markers in depressed subjects [106]. Taken together, these data suggest that PUFAs are beneficial for individuals with diagnosed depressive illness [109]. Some studies even suggested that the efficiency of supplementing *n*-3 PUFAs for the prevention and treatment of depression was higher when the EPA content was higher than that of DHA [99]. Comorbidity between depressive and anxiety disorders is common [110], so the effect of PUFA on anxiety has also aroused interest. However, the number of existing studies in this sense is still small. Recent research has shown that the presence and severity of comorbid depressive and anxiety disorder were associated with lower *n*-3 PUFA levels [100,111]. In addition, low plasma levels of *n*-3 PUFA have been found in patients with social anxiety disorders [112], and it has been associated with a higher likelihood of anxiety in women [113]. The hypothesis that *n*-3 PUFA deficiency is causal in anxiety is supported by studies showing that *n*-3 PUFA supplementation resulted in a reduction in anxiety symptoms in young adults without a diagnosis of anxiety disorder compared with the controls [74]. The results obtained in animal models also supported this idea. That is, dietary EPA-enriched phospholipids (EPA-PL) have been shown to alleviate depression- and anxiety-like behavior induced by chronic stress and LPS in mice [67]. Specifically, the administration of EPA-PL improved depressive-like behavior in CUMS-exposed mice. Furthermore, an increase in the number of entries into the center of the open field test was reported after EPA-PL treatment, indicating an alleviation of anxiety-like behavior in CUMS-exposed mice [67,99]. 

Taken together, the clinical and pre-clinical data described above suggest that VLC *n*-3 PUFAs could exert a positive effect on cognition, mood, impulsivity, or anxiety, among others. Little is yet known about the exact mechanism by which they exert such effects, although many studies suggest that it may be mediated by their anti-inflammatory properties. However, more studies are needed to clarify this hypothesis. 

**Table 2:** Effect of VLC *n*-3 PUFAs on the central nervous system.

Author	Sample	Treatment	Main Outcomes
In vitro study			
(Rey et al., 2016) [51]		BV2 microglial cell	RVsE1 (10 nM) or RVsD1 (10 nM) for 30 min
(Titos et al., 2011) [52]		BV2 microglial cell	DHA (10, 50, and 100 mM) for 18 h or RVsD1 (1, 10, and 100 nM) for 5 h
(Xu et al., 2013) [53]		Microglial cell	RVsE1 pre-treatment (100 ng/mL)
(Fourrier et al., 2017) [54]	BV2 microglial cell	DHA-enriched phosphatidylcholine (30 µM) for 24 h	↓ LPS-induced IL-6 production
(De Smedt-Peyrusse et al., 2008) [55]	BV2 microglial cell	DHA (0.3, 3, 30, or 300 μmol/L) for 24 h	↓ LPS-induced level of IL-1β, IL-6, and TNF-α, ↓ LPS-induced NFκB activation, and ↓ CD14 and TLR4 cell-surface expression
(He et al., 2017) [56]	Primary bovine mammary epithelial cells	DHA (25, 50, and 100 μM) for 3 h	↓ LPS-induced IL-1β, IL-6, and TNF-α mRNA expression
(Zgórzyńska et al., 2021) [57]	Primary rat cortical astrocyte	DHA (10, 30, and 50 μM) for 24 h	↓ Response to IL-1 β, ↓ release of TNFα and IL-6, and ↓ iNOS and COX-2 levels
(Kawashima et al., 2008) [59]	C6 glioma cells	EPA (50 μM) for 24 h	↓ IL-6 production, ↓ IL-6 mRNA expression, and PPAR gamma antagonist abolish the inhibitory effect of EPA
(Inoue et al., 2017) [61]	MG6 and BV2 microglial cells	EPA (200 μM) or DHA (200 μM) for 30 min	↓ LPS-induced IL-6 production
		EPA and DHA (200 μM) for 30 min	Activation of SIRT1 pathway
(Ma et al., 2020) [62]	BV2 microglial cell	DPA (50 μM) for 24 h	↓ mRNA expression of COX2 in amyloid-beta42 oligomer-challenged cells
(Liu et al., 2021) [63]	BV2 microglial cell	DPA (50 μM) for 24 h	↓ LPS-induced IL-1β, TNF-α, and NO mRNA expression
(Tian et al., 2017) [64]	Macrophage-like RAW264.7 cell	DPA, EPA, or DHA (25–75 μM) for 72 h	DPA and DHA ↓ LPS-induced IL-1β, IL-6, iNOS, and COX-2 mRNA expression
Pre-clinical studies			
(Fourrier et al., 2017) [54]	C57BL6/J mice	PC-DHA (4.33 µg/g, iv)	↓ LPS-induced IL-6 mRNA expression in the hippocampus
(Dong et al., 2018) [58]	Long–Evans rats	Diet supplemented with 0.8% EPA for 42 days	↓ GFAP and TNF-α mRNA expression induced by IL-1β in the hippocampus and ↑ hippocampal BDNF mRNA expression
(Ma et al., 2020) [62]	E3FAD and E4FAD mice	DPA (700 mg/kg, oral gavage) for 3 weeks	↑ DPA level in the brain, ↓ brain pro-inflammatory cytokines IL-6 and TNF-α and ↓IL-10, and ↓ COX gene expression
(Delpech et al., 2014) [66]	Fat-1 transgenic mice	--------	↑ DPA and EPA level in the hippocampus of KO mice, LPS Impairs spatial memory in WT but not in KO mice, and ↓ LPS-induced IL-1β mRNA expression compared with WT mice
(Wang et al., 2021) [67]	ICR mice	Diet supplemented with 0.6% EPA for 4 weeks	↓ IL-1β mRNA expression in the hippocampus, thalamus, and cortex, improve anxiety- and depression-like behavior induced by chronic stress, and attenuate reduction in DA, 5-HT, and NE induced by chronic stress
(Patel et al., 2020) [68]	Pups Sprague–Dawley rats	Stearidonic-acid-enriched maternal diet (3%) 5 days prior to parturition and through the suckling period.	↑ Proportion of EPA, DPA, and DHA in plasma phospholipid and ↓ LPS-induced IL-6 and TNF-α production
(Labrousse et al., 2012) [69]	Aged C57Bl6/J mice (20 months)	Diet supplemented with 10% EPA and 7% DHA for 60 days	↑ DHA and EPA levels in the brain, ↓ IL-6 and TNF-α mRNA expression in the hippocampus, ↓IL-1β expression in plasm, and restored spatial memory deficits associated with age
(Kelly et al., 2011) [70]	Aged rats (20–22 months)	DPA (200 mg/kg) for 56 days	↓ Escape latency and mean latency in an MWM compared with the aged control group and ↓ microglial activation, caspase-3n and oxidative stress
(Jost et al., 2022) [79]	Rats	10% PUFA-enriched diet for 5 weeks	↓ Number of trials to reach the learning criterion in the T-maze
(Lamontagne-Kam et al., 2023) [80]	Sprague–Dawley rats	2.1% DHA-enriched diet for 47 days	DHA-fed females ↑ % time in the correct quadrant in MWM than control groups
(Hauser et al., 2018) [91]	Wistar rats	Transgenerational exposure to *n*-3 PUFA-deficient diet	↓ % of correct responses and ↑% omission on 3CSRTT, ↑ premature responses and more timeout responses on 3CSRTT, and impaired performances was partly corrected by *n*-3 PUFA-sufficient diet
(Dervola et al., 2012) [92]	Spontaneously hypertensive rodents (SHR/NCrl)	EPA, 300 mg/g; DHA, 190 mg/g for 40 days	↑ % responses on the reinforcer-producing level in males, ↓ premature responses and lever-directed hyperactivity in males, and ↑ DA and 5-HT turnover in males
Randomized clinical trial			
(Bo et al., 2017) [73]	Elderly with MCI	720 mg/day EPA and 480 mg/day DHA for 6 months	↑ Total score of the Basic Cognitive Aptitude Tests, ↑ WM in men but not in women, and ↓ IL-6 and TNF-α levels in plasma
(Kiecolt-Glaser et al., 2011) [74]	Healthy young adults	348 mg/day DHA and 2085 mg/day EPA for 4 months	↓ Anxiety symptoms, no significant effect in depressive symptoms, and ↓ LPS-stimulated IL-6 and TNF- α production
(Kiecolt-Glaser et al., 2012) [75]	Healthy adults	348 mg/day DHA and 2085 mg/day EPA for 4 months	↓ Serum levels of IL-6 and TNF-α and no significant difference in depressive symptoms
(Khalili et al., 2021) [76]	Patients with type-2 diabetes mellitus	1000–2000 mg of *n*-3 PUFA for 3 months	↓ TNF-α and c-reactive protein levels
(Lee et al., 2013) [85]	Participants with MCI	1290 mg/day DHA and 450 mg/day EPA for 12 months	↑ Short-term and working memory
(Nilsson et al., 2012) [86]	Healthy adults	1050 mg/day DHA and 1500 mg/day EPA for 5 weeks	Better performance in WM test
(Nolan et al., 2022) [87]	Patients with mild–moderate AD	150 mg/day EPA and 500 mg/day DHA for 12 months	↑ Mood and memory
(Jackson et al., 2016) [88]	Healthy older adult	128 mg/day EPA and 896 mg/day DHA for 6 months	No effect on performance of the CDB tasks
(Phillips et al., 2015) [89]	Individuals with cognitive impairment, no dementia	600 mg/day EPA and 625 mg/day DHA for 4 months)	No effect on MMSE and HVLT-R score
(Anand and Sachdeva, 2016) [93]	ADHD children (4–8 years)	180 mg/day EPA and 180 mg/day DHA for 4 months	↓ Conners’ ADHD scores and ↓ hyperactive and impulsive symptoms
(Bos et al., 2015) [94]	ADHD children (8–14 years)	65 mg/day EPA and 650 mg/day DHA for 4 months	↑ scores on CBCL attention problems subscale and no significant effects on the Rule Breaking and Aggressive Behavior subscales
(San Mauro Martin et al., 2022) [95]	ADHD children (6–16 years)	550 mg/day EPA and 225 mg/day DHA for 8 weeks	↓ BIS-11c total score

↓: decrease; ↑: increase; 3-CSRTT: 3-choice serial reaction-time task; 5-HT: serotonin; ADHD: attention-deficit hyperactivity disorder; BIS-11c: Barrat Impulsiveness Scale for Children; CBCL: child behavior checklist; CDB: cognitive demand battery; DA: dopamine; HVLT-R: Hopkins Verbal Learning Test–revised; IL: interleukin; KO: knock-out; LPS: lipopolisacharide; MCI: mild cognitive impairment; MMSE: mini-mental state examination; MWM: Morris water maze; NE: norepinephrine; TNF: tumor necrosis factor; WM: working memory; WT: wild-type.

## 5. Role of VLC *n*-3 PUFAs on Alcohol Use Disorders

Alcohol use disorder (AUD) is a highly prevalent mental health condition [1] characterized by continued alcohol use despite negative consequences [114]. Although heavy drinking does not automatically indicate AUD, it is important to note that heavy drinkers have a significantly higher risk of developing it [115]. Chronic and excessive alcohol consumption is associated with various health problems, such as cardiovascular disease, liver cirrhosis, and cancer. Furthermore, it has been linked to a wide range of effects on the CNS, including behavioral and cognitive impairment.

**Figure 3:**
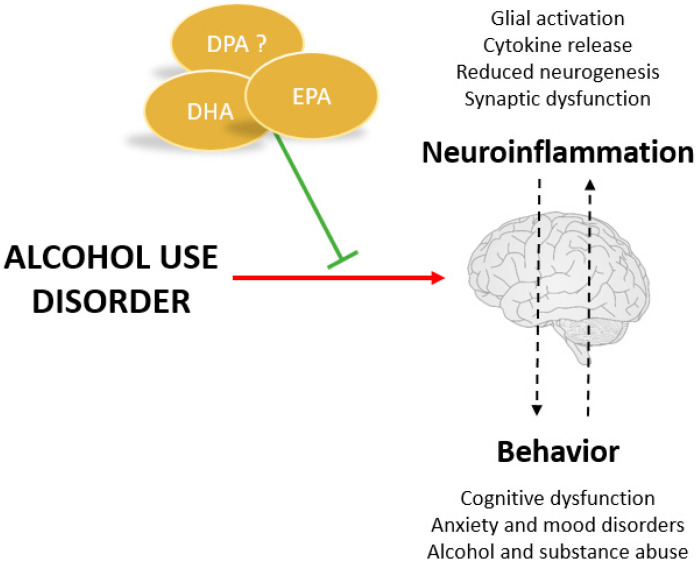
Potential benefits of VLC *n*-3 PUFAs for treating alcohol use disorders.

There is still insufficient evidence to demonstrate the therapeutic potential of VLC *n*-3 PUFAs in drug addiction and, specifically, in alcohol abuse. However, numerous studies have emerged in recent years that already pointed to the fact that they may play an important role in the prevention of withdrawal symptoms and relapse. As shown in Figure 3, one of the main hypotheses is that they exert this effect through their anti-inflammatory role and their effect on synaptic remodelling [13]. As mentioned above, increasing the amount of VLC *n*-3 PUFAs in the diet can reduce LPS-induced neuroinflammatory responses and is capable of restoring synaptic structure and transmission in both neurons and microglia. Existing studies on the effect of VLC *n*-3 PUFAs on alcohol consumption and related behaviors are detailed below.

### 5.1. VLC n-3 PUFAs and Alcohol-Induced Brain Damage

Alcohol can cause brain damage and, in some cases, leads to neurodegeneration [10]. Studies have shown that alcohol promotes long-term increase in pro-inflammatory cytokine levels and oxidative stress, creating a neurotoxic environment that can lead to brain damage [116,117]. Upregulated hippocampal IL-1β and IL-6 expression, as well as phosphorylation of tau proteins, have been implicated in cognitive deficits associated with chronic alcohol exposure by mediating the synaptic dysfunction [118].

Genetic and pharmacological studies have indicated that neuroimmune signalling plays a crucial role in the regulation of alcohol consumption in rodents [10]. Ethanol exposure affects the neuroimmune system, leading to changes in the mRNA levels of the pro-inflammatory cytokines (TNF-α, IL-6, and CCL2) in rodents [119]. In humans, an acute alcohol challenge significantly increases the IL-8 and TNF-α levels [120]. Furthermore, binge-like alcohol exposure activates astrocytes and increases GFAP immunoreactivity [121] while decreasing IL-10 immunoreactivity [122]. These studies suggest that the ethanol-induced disruption in neuroimmune signalling may modulate excessive ethanol intake. In particular, ethanol consumption was reduced by bilateral infusions of an IL-1 receptor antagonist (IL-1Ra) into the basolateral amygdala [123]. On the contrary, administration of LPS, an immune activator, increased ethanol consumption in mice while blocking the neurokinin-1 receptor (NK1R), which attenuated the neuroinflammation and counteracted the effect of LPS on ethanol consumption [124,125]. Furthermore, the administration of anti-inflammatory compounds, such as the PPAR agonist fenofibrate and pioglitazone, reduced ethanol drinking in rats [126,127]. Consistent with pharmacological studies, genetic deletion of IL-6, TLR2, or CD14 diminished the ethanol consumption [124,128]. These findings suggest that targeting the neuroinflammation could be a potential treatment option for alcohol use disorder. Taking into account the anti-inflammatory effect triggered by VLC *n*-3 PUFAs, as discussed in the previous section, they may represent suitable candidates for nutraceutical supplementation in the treatment of excessive alcohol consumption, along with the traditional pharmacological approach.

Alcohol-induced neuroinflammation is closely associated with neurodegeneration, as an excessive or prolonged inflammatory response to alcohol can lead to apoptosis in neurons and glia [10,122] as well as inhibit neuronal plasticity and neurogenesis [129,130,131]. In humans, heavy alcohol consumption is associated with significant brain shrinkage, loss of hippocampal and frontal lobe volume, and white matter atrophy (for a review, see [132]). Binge drinking also negatively affects adult hippocampal neurogenesis [130,131] and neural stem cell proliferation [133,134]. External factors, such as environmental enrichment or physical exercise, have been shown to stimulate neurogenesis [135,136]. Evidence suggests that EPA and DHA enhance the expression of BDNF and GDNF, neurotrophic factors implicated in proliferation, differentiation, and neuronal survival [137]. On the contrary, the depletion of brain DHA could underlie aberrant neuroapoptosis in fetal alcoholism models [138]. In this regard, it is currently suggested that supplementation with VLC *n*-3 PUFAs could protect or even reverse alcohol-induced brain damage. Previous studies have shown that treatment with fish oil, which is rich in VLC *n*-3 PUFA, can reverse chronic alcohol-exposure-induced synaptic changes in the nucleus accumbens and alleviate withdrawal symptoms [139]. Furthermore, postnatal supplementation with VLC *n*-3 PUFAs has been found to reduce long-term deficits in hippocampal synaptic plasticity caused by prenatal ethanol exposure [140]. Moreover, N-docosahexenoyethanolamine, a metabolite of DHA, has been demonstrated to reverse alcohol-impaired neural stem cell differentiation [141]. Finally, DHA supplementation has been shown to significantly improve neurodegeneration induced by binge alcohol exposure in the cultured brain slices of adult rats [142]. Evidence suggests that supplementation with VLC *n*-3 PUFAs may be a rational approach to potentially reduce alcohol-related damage to the nervous system and has the potential to mitigate the teratogenic effects of alcohol.

Neuroinflammation and oxidative stress are closely interconnected, as neuroinflammation can induce oxidative stress, and oxidative stress can, in turn, activate the inflammatory signalling pathways [143]. Alcohol consumption promotes oxidative stress, which is characterized by an imbalance between the production of free radicals, such as reactive oxygen species (ROS), and compromised or insufficient antioxidant defence response [144]. Pre-clinical studies have shown that chronic ethanol intake induces oxidative stress [117] and reduces the level of endogenous antioxidants, such as glutathione or GPX [145]. Similar findings were observed in alcoholic patients, where elevated levels of malondialdehyde and reduced superoxide dismutase activity were observed [146]. These parameters of oxidative stress were associated with the severity of alcohol dependence and the risk of drinking relapse [147]. Considering the anti-inflammatory effect of VLC *n*-3 PUFAs, their potential efficacy in controlling the oxidative stress associated with neuroinflammation has been investigated. Although studies on this topic are limited, existing evidence suggests the beneficial effects of VLC *n*-3 PUFAs on adipose tissue inflammation and oxidative stress [148,149]. Dietary enrichment with DHA has been shown to reduce hippocampal ROS in a rat model of beta-amyloid-induced toxicity [149]. DHA also counteracts alcohol-induced changes in the oxidative stress cascade, including activation of phospholipase A2 and alteration of aquaporin, thus exerting a positive effect on oxidative stress [150]. However, it should be noted that some studies have reported an increase in oxidative stress with VLC *n*-3 PUFA supplementation in both humans and animals [151,152]. Therefore, more research is needed to determine the potential antioxidative effect of PUFA and its impact on alcohol-induced oxidative stress.

The detrimental effect of alcohol on the brain phospholipid composition, especially PUFA, is evident. Long-term ethanol exposure has been shown to lower DHA-containing phospholipid species in neuronal tissues [138]. A case–control study of chronic alcoholics revealed DHA metabolic deficits in the brain [153]. Clinical data also indicate a low DHA concentration in plasma and erythrocytes in pregnant women who consume alcohol [154]. Heavy drinkers with reduced VLC *n*-3 PUFA levels are associated with the occurrence of liver injury in women [155] and patients with comorbid hepatitis C [156]. As previously mentioned, VLC *n*-3 PUFAs have beneficial effects in resolving inflammation and oxidative stress, and they can positively modulate alcohol-induced injuries not only in the brain but also in other organs, such as the liver.

Alcohol-induced alterations in the neuroimmune system can modulate several neurotransmitter systems that are important in addiction [157]. VLC *n*-3 PUFAs can also engage various neurotransmission systems, particularly dopaminergic and serotonergic, that are involved in many aspects of motivated behaviour, including reward processing. Dietary consumption rich in VLC *n*-3 PUFAs has been shown to increase the striatal turnover of dopamine and serotonin [92]. However, the deficiency of VLC *n*-3 PUFAs leads to a reduced number of dopaminergic neurons in the substantia nigra and ventral tegmental area [158]. In a rat model of *n*-3 PUFA deficiency, DHA supplementation restored altered dopamine and serotonin release and suggested that the mesolimbic dopamine pathway may be less active in VLC *n*-3 PUFA-deficient animals [159]. Therefore, modulation of the dopaminergic system by VLC *n*-3 PUFAs may affect motivation and reward response and, subsequently, alcohol consumption.

In conclusion, the results presented here strongly suggest that VLC *n*-3 PUFAs, through various mechanisms, could play a promising protective role in counteracting alcohol-induced brain damage.

### 5.2. VLC n-3 PUFAs and Cognitive and Behavioral Impairments Associated with Alcohol Consumption

Brain damage associated with chronic alcohol use also appears to be related to impaired cognitive functioning and behavioral patterns that persist into abstinence. Alcohol-induced brain damage has even been proposed to accelerate the brain aging process. Thus, heavy alcohol consumption (5+ drinks per day) is hypothesized to be associated with accelerated cognitive aging, and those who are current heavy drinkers exhibited the greatest cognitive deficits [160].

As suggested by pre-clinical and clinical evidence, chronic alcohol consumption persistently changes neurobiology, increasing risky decision-making and affecting reflection impulsivity [161]. Alcohol use also significantly influences affective behaviors [162]. According to previous studies, the co-occurrence of AUD and depressive disorders is associated with a greater severity and a worse prognosis for both disorders [163]. Similarly, animal studies have shown that alcohol causes anxiety and depression-like behavior in adult rats [117,164]. In addition, higher levels of a negative effect have specifically been associated with the initiation and relapse of alcohol use disorder [165]. Therefore, interventions that focus on the reduction of anxiety and the improvement of emotional state are important in patients with AUD to reduce alcohol consumption and prevent relapse. VLC *n*-3 PUFAs have consistently shown beneficial effects on mood disorders in pre-clinical and clinical trials [13,67,100,101,104]. However, there is a lack of research directly addressing the preventive effect of PUFAs on anxiety in patients with AUD or heavy drinking. Some pre-clinical studies have shown that DHA supplementation reduces ethanol-induced hyperlocomotion and anxiety-like behavior [166]. Furthermore, DHA supplementation has been found to ameliorate deficits in social behavior and ultrasonic vocalizations, which are measures of behavioral anxiety, in rodents exposed to ethanol during prenatal development [167]. Furthermore, there is evidence of an association between PUFA supplementation and decreased anxiety and anger among substance users [168]. Further research is needed to explore the potential preventive effects of PUFAs on anxiety in people with AUD or heavy drinking.

Pre-clinical data showed that binge-like ethanol exposure during adolescence altered the prefrontal cortex (PFC) and hippocampus-dependent functions [169,170]. Similarly, adolescents who exhibited a binge drinking pattern showed deficits in spatial working memory and executive functioning [171,172]. As shown earlier, PUFAs could exert a positive effect on ameliorating the memory [69,73,79,86] and can reduce alcohol-related damage to the hippocampus and the PFC [140,142].

Levels of VLC *n*-3 PUFAs may have possible clinical implications for alcohol-related outcomes. A low level of *n*-3 PUFA was associated with an increased risk of relapse or study dropout in treatment-seeking substance abusers [173] and with lower alcohol consumption in non-alcoholic people in the IMMIDIET study [174]. Recent work found an association between the plasma level of VLC *n*-3 PUFA and the initial alcohol sensitivity in a population-based cohort of adolescents, where a higher alcohol sensitivity was related to higher VLC *n*-3 PUFA levels [175]. It has even been proposed that variation in the genes regulating the VLC *n*-3 PUFA level was significantly associated with ethanol-related phenotypes. Interestingly, the *GCKR*, *FADS2*, and *ACOX1* genes showed a significant association with alcohol consumption, and the *GCKR* gene was associated with AUD [176].

Alcohol consumption was associated with the concentration of VLC *n*-3 PUFA in the blood according to pre-clinical and clinical data. The IMMIDIET project found that alcohol consumption was associated with higher levels of EPA and DHA in the blood of women, and with higher EPA levels in men [174]. An altered fatty acid profile, with decreased DHA and increased EPA levels, was also observed in mothers with heavy alcohol consumption who had infants diagnosed with Fetal Alcohol Spectrum Disorder [154]. Finally, genetic studies in rodents have shown that a diet high in VLC *n*-3 PUFA, with a higher concentration of EPA than DHA, increased voluntary alcohol consumption in C57BL/6J (with a high alcohol preference) but not in DBA/2J mice (with a low alcohol preference); moreover, it also modulated alcohol metabolism and alcohol-induced locomotor activity [177]. On the other hand, a diet rich in DHA reduced voluntary alcohol consumption in P rats showing a high alcohol preference [103]. Furthermore, VLC *n*-3 PUFAs appeared to play an important role in preventing relapse and alleviating withdrawal symptoms after chronic alcohol exposure. A diet rich in VLC *n*-3 PUFAs decreased the propensity to relapse in adult mice chronically exposed to ethanol, reducing the psychomotor stimulant response to alcohol after a 7-day withdrawal and decreasing the ethanol-induced place preference [139]. Finally, the efficacy of VLC *n*-3 PUFA supplementation in reducing the anxiety and cortisol basal levels in abstinent alcoholics undergoing a residential rehabilitation program was demonstrated [178].

Although studies are still scarce, especially those using only DPA, there is promising evidence suggesting the potential of VLC *n*-3 PUFAs to modulate heavy drinking and its associated cognitive and behavioral impairments. However, further research is needed to address the existing variability among the studies. Specifically, exploring how factors such as gender or age of the participants could influence these results will be challenges to consider.

## 6. Conclusions

Several clinical and epidemiological studies have highlighted the many health benefits of VLC *n*-3 PUFAs, ranging from cardiovascular disease to several psychiatric disorders. As mentioned above, VLC *n*-3 PUFA may provide its beneficial effect by acting as an anti-inflammatory, anti-oxidative stress, or anti-neurodegeneration agent, among others.

VLC *n*-3 PUFAs are currently attractive candidates for the treatment of alcohol use disorders. Both DHA and EPA can modulate alcohol-induced neurobiological changes through several mechanisms related to their anti-inflammatory and antioxidant effect and their role in the regulation of neurotransmitter systems. Although DPA shows potential as well, further studies are still needed to fully understand its efficacy. Additionally, it is important to note that VLC *n*-3 PUFA has the added advantage of being safe and well tolerated, with no severe adverse effects.

Although there is promising evidence suggesting the potential of VLC *n*-3 PUFAs to modulate alcohol-induced brain damage and its associated cognitive and behavioral impairments, more research is needed to assess the optimal conditions for their effectiveness. This includes investigating factors such as dosage, route of administration, duration of exposure, or specific type of PUFA, as well as other variables. Additionally, it is essential to gain a deeper understanding of the underlying mechanisms through which VLC *n*-3 PUFAs exert their beneficial effects. Diet-based interventions on animal models can play a vital role in achieving this goal. Conducting more comprehensive studies will help to elucidate the specific conditions and mechanism by which PUFAs can effectively mitigate alcohol-related damage and improve the results in individuals with alcohol use disorders or heavy drinking.

## Figures and Tables

**Table 1:** Proportions of EPA, DPA, and DHA in the lipid fraction of marine sources.

Lipid Source	VLC *n*-3 PUFAs (% of Total FAs)			Reference
	EPA	DPA	DHA	
Fishes				
Cod flesh	19	2	33	[15]
Cod liver	12	2	13	[15]
Flounder	15	3	19	[15]
Haddock	15	2	25	[15]
Halibut	10	3	31	[15]
Horse mackerel	7–9	2–3	30–32	[33]
Menhaden	18	2	10	[15]
Salmon (farmed)	6	3	8	[34]
Salmon (wild)	7	3	13	[34]
Sardine	10–14	1–2	15–27	[35,36]
Seabream	5–11	4	18–32	[33]
Tuna	2–12	1–2	12–28	[36,37]
Marine mammals				
Seal	4–11	4–5	7–26	[15,38]
Crustaceans				
Krill	18	<1	12	[39]
Lobster	11–17	1	8–11	[15,40]
Red crab	12	2	12	[15]
Rock crab	21	2	10	[15]
Shrimp	15–17	1	11–13	[15,40]
Bivalves				
Blue mussel	20	-	13	[15]
Icelandic scallop	27	-	26	[15]
Surf clam	23	-	14	[15]
Cephalopods				
Common octopus	16	1–2	21–28	[15,41]
Squid	14–16	1	17–37	[15,41]

## Data Availability

Not applicable.
